# Safety, feasibility and functionality of activated autologous dendritic cells for intratumoral injection in solid tumors: a Phase I clinical trial

**DOI:** 10.1186/2051-1426-3-S2-P199

**Published:** 2015-11-04

**Authors:** Vivek Subbiah, Ravi Murthy, David Hong, Robert Prins, Chitra Hosing, Robert Brown, McGuire Mary, Aung Naing, Siquing Fu, Anthony Conley, Indreshpaul Kaur, Kyle Hendricks, Deepthi Kolli, Lori Noffsinger, Marnix Bosch

**Affiliations:** 1 U.T.M.D. Anderson Cancer Center, Houston, TX, USA; 2 The University of Texas MD Anderson Cancer Center, Houston, TX, USA; 3 UCLA Neurosurgery, Los Angeles, CA, USA; 4 UT Health, University of Texas Health Science center at Houston, Houston, TX, USA; 5 Cognate Bioservices, Inc, Memphis, TN, USA; 6 Cognate Bioservices, Inc, Baltimore, MD, USA; 7 Northwest Biotherapeutics, Bethesda, MD, USA

## Background

Dendritic cells (DC) are proficient in initiating adaptive immune responses, through the uptake and subsequent presentation to the immune system of antigenic compounds. In preclinical studies, activated DC (aDC; DCVax®-Direct) were shown to be superior to immature DC in clearing tumors from mice, upon intratumoral injection.

## Methods

Forty patients were enrolled in a Phase I dose escalation trial to test the safety and feasibility of intratumoral injection of aDC in solid tumors. aDC were administered intratumorally under image guidance, at a dose of 2 million, 6 million, or 15 million live, activated, autologous DC per injection. At each injection visit (days 0, 7, 14, then weeks 8, 16 and 32), a single lesion was injected. Biopsies were assessed for tumor necrosis and for infiltrating lymphocytes. Tumor size was monitored through standard imaging procedures, and blood was collected for immune monitoring. The aDC were assessed for expression of co-stimulatory molecules and for the production of cytokines.

## Results

Intratumoral injection under image guidance was generally well tolerated and feasible. In total, 149 i.t. injections were performed, in 17 patients at the 2 million, 20 at the 6 million, and 3 at the 15 million dose level, with mild to moderate fevers as the most frequently observed adverse events. Biopsies of the injected tumors showed appearance of tumor necrosis in 62%, and T cell infiltrates in 54%. Stabilization of disease was found to correlate with survival, and with a specific cytokine profile of the aDC which is consistent with induction of Th-1 type immune responses.

## Conclusions

Intratumoral injection of autologous, activated DC is feasible without significant toxicity in multiple solid tumors, and can elicit local and systemic immune responses. Specific characteristics of the injected dendritic cells may predict tumor response and survival.

## Trial registration

ClinicalTrials.gov identifier NCT01882946.

